# Perspectives in Biophotonics: a special issue in honor of the International Graduate Summer School on Biophotonics

**DOI:** 10.1117/1.JBO.27.5.050102

**Published:** 2022-05-23

**Authors:** Stefan Andersson-Engels, Peter E. Andersen

**Affiliations:** a Biophotonics@Tyndall, IPIC, Tyndall National Institute, Ireland; b University College Cork, Department of Physics, Ireland; c Technical University of Denmark, Denmark

## Abstract

The guest editorial introduces the Journal of Biomedical Optics special collection, Perspectives in Biophotonics, https://www.spiedigitallibrary.org/biophotonics-tutorials.

Tutorials from lecturers and organizers of the International Graduate Summer School on Biophotonics^1^ have been given biannually since 2003 on the island Ven in Sweden. In honor of the 10th edition of the summer school, JBO has published a virtual special issue “Perspectives in Biophotonics,” featuring updates to those tutorials as perspective articles: https://www.spiedigitallibrary.org/biophotonics-tutorials. This special issue is meant to complement the large number of tutorial and review papers in previous special sections in JBO from past schools and also be forward-looking to highlight opportunities and unmet needs to consider for researchers in our field. This special issue came out of the Covid pandemic postponing the 10th summer school in this series. The global pandemic lockdowns made us reach out to the lecturers at the school to ask whether they were willing to write a perspective paper in their field of expertise for this special issue in JBO. These ten perspective papers will hopefully serve the field well and will become preparation reading for the participants appropriate for the upcoming 10th Biophotonics School.

## 1 Motivation and Purpose of Biophotonics Graduate Schools

The purpose of the International Graduate Summer School Biophotonics is to provide education for postgraduate students at the highest international level. The school has so far always experienced higher interest than the number of students we can accommodate, illustrating the needs it fills in the field, and we have therefore implemented a peer-review selection procedure. Apart from learning from renowned lecturers and scientists, the international atmosphere in having about 80 biophotonics scientists from across the world for a week in a confined space, on the beautiful small island Ven, makes networking opportunities phenomenally good. The school certainly has the potential to create lifelong friendships, and to help advance the field by exchange of ideas. Lecturers are invited to teach at the school based on their scientific merits and pedagogical skills and they reflect an international and diverse scientific community of highest quality. They are all encouraged to stay for a minimum of four days during the week-long school to facilitate informal discussions with participants around their lectures, expertise, and experiences. Students are requested to present their research in poster sessions early in the school week, providing ample opportunities to get to know all participants and their research. Lectures are scheduled daily before lunch and after dinner, providing afternoons for sight-seeing around the island, visiting the museum of the famous astronomer Tycho Brahe (1546–1601), sports activities, or smaller workshop on specified topics (e.g., general topics such as EDI or career opportunities, technical topics such as Monte Carlo simulations, or clinical perspective topics). This format sets a busy schedule during the week, while also allowing for informal discussions during the school.

**Figure:**
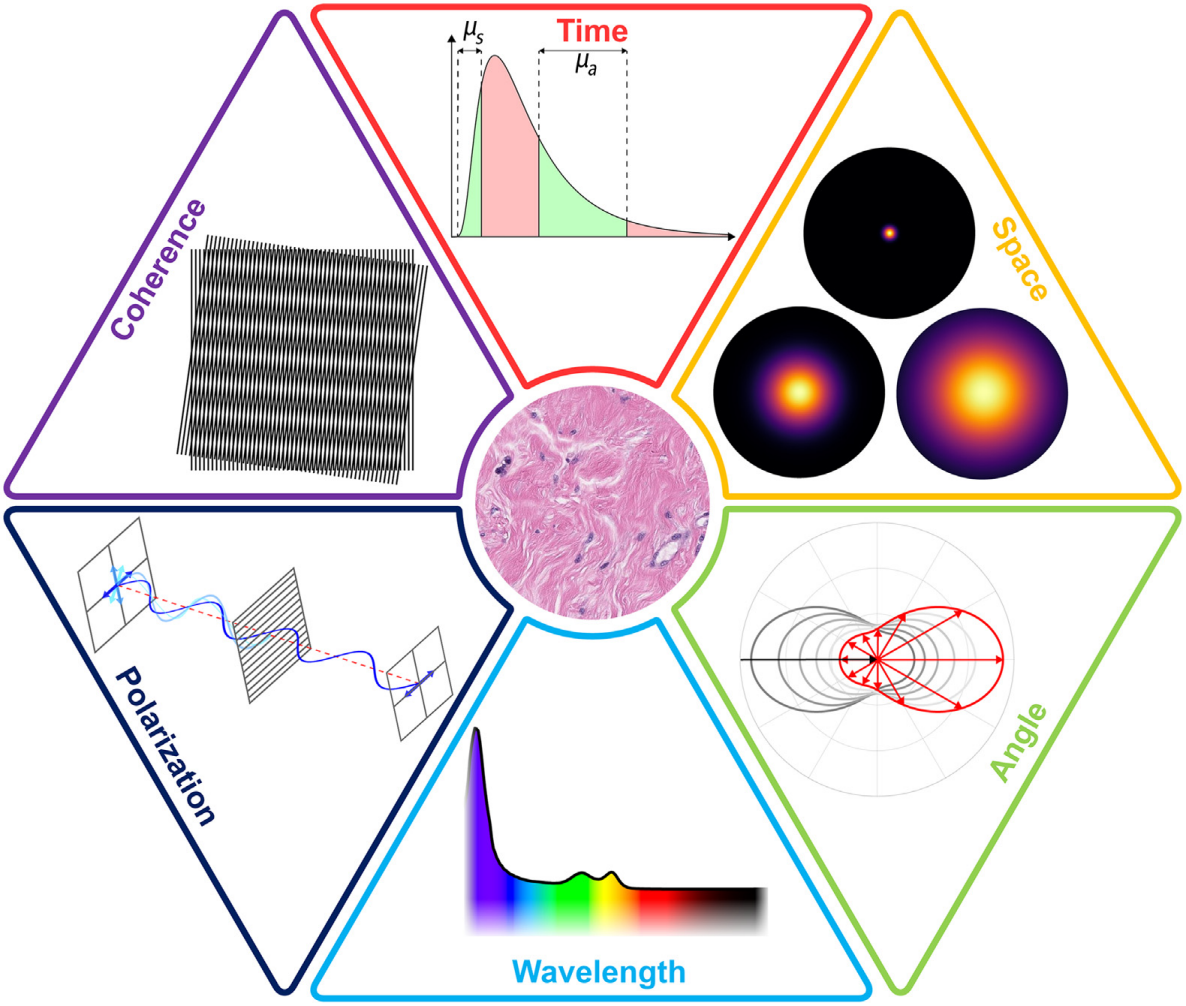
The JBO special issue, Perspectives in Biophotonics, is available at https://www.spiedigitallibrary.org/biophotonics-tutorials. Image credit: Streeter, Jacques, and Pogue, doi 10.1117/1.JBO.26.7.070601

This special issue comprises ten perspective papers covering a wide variety of biophotonics techniques and applications. A relatively new field in biophotonics is guidance of orthopedic procedures. Emerging optical sensing promises fewer side effects with new, more effective approaches aimed at improving patient outcomes following orthopedic surgery. Fisher et al. provide a comprehensive overview with an opinionated perspective of this field (https://doi.org/10.1117/1.JBO.27.1.010601). The applications focused on in this paper are total hip arthroplasty, clavicle fracture fixation, pedicle screw placement, and lumbar decompression procedures; it also discusses integration of optical devices into orthopaedic tools.

Novel technologies that can further boost the optical coherence tomography (OCT) imaging area are discussed by Leitgeb et al. (https://doi.org/10.1117/1.JBO.26.10.100601). OCT has already 75,000 publications and has been the fastest growing imaging modality with substantial clinical and economic impacts and acceptance. This comprehensive review is a follow up of the previous review of the field (https://doi.org/10.1117/1.JBO.19.7.071412), and identifies further potential improvements in high resolution and speed, multimodality implementation and the use of novel contrast agents and functions to explore metabolic signals.

Another interesting technique based on light coherence is speckle imaging. Hajjarian and Nadkarni describe their perspective on how this technique can be employed to measure micro-rheology of importance for understanding mechano-biological aspects of malignant tumours (https://doi.org/10.1117/1.JBO.26.9.090601). This is a perspective extension of their recent review of the field (https://doi.org/10.1117/1.JBO.25.5.050801).

The unique specificity of Raman spectroscopy (RS) to identify and measure biomolecules is well established (https://doi.org/10.1117/1.JBO.23.7.071210), as is the low acquisition rate and lack of direct depth sensitivity. Schie et al. provide their expert view of how Raman spectroscopy can be used in multimodality approaches to complement other, faster and less specific modalities (https://doi.org/10.1117/1.JBO.26.8.080601). Their view is clear: they strongly believe RS has a key role in a multimodality-approach for clinical translation of biophotonics-based diagnostics.

Light diffusion is found in many applications in biophotonics. Streeter at al. discuss a brief, unifying analytical framework to describe the transition from light delivered to diffuse light transport in tissue (https://doi.org/10.1117/1.JBO.26.7.070601). Sub-populations of photons are considered to interpret this transition. This paper is an extension of the previously published review paper in the field (https://doi.org/10.1117/1.2967535).

The concept of optical forces is being increasingly employed for manipulation in biology and has facilitated exceptional advances and breakthroughs. Arthur Ashkin was awarded the Nobel Prize in Physics 2018 for his seminal contribution to this field. On this background, Corsetti and Dholakia describe the forces generated in optical tweezers and acoustic traps, complement a previous review of the field (https://doi.org/10.1117/1.3475958), and insightfully discuss potential future impact on biology of optical manipulation (https://doi.org/10.1117/1.JBO.26.7.070602).

Fluorescence lifetime imaging (FLIM) has become a powerful tool to image molecular interactions in tissue. Datta et al. provide updates of impactful innovations in FLIM (https://doi.org/10.1117/1.JBO.26.7.070603). In their perspective paper they cover advances in instrumentation and data analysis as well as novel application approaches. Their presentation complements a previous recent comprehensive review of the field (https://doi.org/10.1117/1.JBO.25.7.071203).

Photodynamic therapy is first line treatment options for certain conditions, while it is still a treatment under evaluation for other lesions. Komolibus et al. provide their perspective on how interstitial photodynamic therapy (IPDT) may develop, and in particularly for malignant diseases in the central nervous system, prostate gland, breast, and head and neck region (https://doi.org/10.1117/1.JBO.26.7.070604). They analyze the need for improved therapeutic tools in these areas and discuss how IPDT can fill these unmet needs. A review of IPDT was previously published (https://doi.org/10.1117/1.3466579).

Prince and Potma provide an analysis of the potential of coherent Raman scattering microscopy, how it has developed from an exotic technique in the early 1980s to now have the ability to conduct a time-lapses of dynamic processes and 3D scan of tissues at similar rates as fluorescence (https://doi.org/10.1117/1.JBO.26.6.060601). This paper complements the previously published tutorial (https://doi.org/10.1117/1.JBO.19.7.071407), and focuses on diagnostic capabilities of the technique in terms of specificity, sensitivity, and speed.

Yao and Wang complement the tutorial from 2016 (https://doi.org/10.1117/1.JBO.21.6.061007) with a perspective focusing primarily on the recent photoacoustic tomography innovations in volumetric deep-tissue imaging, high-speed wide-field microscopic imaging, high-sensitivity optical ultrasound detection, and machine-learning enhanced image reconstruction and data processing (https://doi.org/10.1117/1.JBO.26.6.060602). They provide examples how these advances will be useful for several specific applications.

Other tutorial papers published in connection to previous special sections organized from the Biophotonics summer school include Sevick-Muraca and Rasmussen, on molecular imaging with optics (https://doi.org/10.1117/1.2953185), O’Sullivan et al., on diffuse optical imaging (https://doi.org/10.1117/1.JBO.17.7.071311), and Taylor on fiber-based sources for biophotonic applications (https://doi:10.1117/1.JBO.21.6.061010).

We believe and hope these perspective papers will serve the field well. They may serve as useful inspiration of new research as well as stimulate new ideas and talents in the field in a foreseeable future.
